# Miscellany

**Published:** 1910-01

**Authors:** 


### MISCELLANY.

A board of commissioned medical officers will be convened to meet at the Bureau of Public Health and Marine-Hospital Service, 3 B street SE., Washington, D. C., Monday, January 24, 1910, at 10 o'clock a. m., for the purpose of examining candidates for admission to the grade of assistant surgeon in the Public Health and Marine-Hospital Service. Candidates must be between 22 and 30 years of age, graduates of a reputable medical college, and must furnish testimonials from responsible persons as to their professional and moral character.

Assistant surgeons receive $1,600, passed assistant surgeons $2,000, and surgeons $2,500 a year. Officers are entitled to furnished quarters for themselves and their families, or, at stations where quarters can not be provided, they receive commutation at the rate of $30.00, $40.00, and $50.00 a month, according to grade. All grades above that of assistant surgeon receive longevity pay, 10 per cent. in addition to the regular salary for every five years' service up to 40 per cent. after twenty years' service. The tenure of office is permanent. Officers traveling under orders are allowed actual expenses.

For further information, or for invitation to appear before the board of examiners, address "Surgeon-General, Public Health and Marine-Hospital Service, Washington, D. C."

---

Civil Service Examinations for the State and County Service.—The State Civil Service Commission will hold examinations on January 22, 1910, for the following positions in which physicians are interested in a greater or less degree: Assistant Bacteriologist, Department of Health, $900 to $1,200; Assistant Superintendent, Monroe County Almshouse Hospital, $600 and maintenance. Women residents of Monroe County only. City Physician, Rochester, N. Y., under Monroe County Superintendent of the Poor, $200 per annum; Director of Laboratories, Department of Health, $3,000 per annum; Photographic Assistant, Pathological Institute, New York City, $720. Men only. Physician (Homeopathic), Physician (Regular), $900 to $1,200; Resident Medical Superintendent, Monroe County Almshouse, $1,000 to $1,200 and maintenance. Men only. Trained Nurse, State Institutions, men and women, $420 to $600 and maintenance; Woman Physician, State Hospitals and Institutions, $1,000 and maintenance. Vacancy at Craig Colony. Applications should be filed on or before January 15. For detailed circular and application blank, address State Civil Service Commission, Albany, N. Y.

